# Essential Role of MFG-E8 for Phagocytic Properties of Microglial Cells

**DOI:** 10.1371/journal.pone.0055754

**Published:** 2013-02-06

**Authors:** Yong Liu, Xuesen Yang, Chenying Guo, Pan Nie, Yan Liu, Jie Ma

**Affiliations:** 1 Southwest Hospital, Southwest Eye Hospital, Third Military Medical University, Chongqing, China; 2 Institute of Tropical Medicine, Third Military Medical University, Chongqing, China; 3 Massachusetts Eye and Ear Infirmary, Department of Ophthalmology, Schepens Eye Research Institute, Harvard Medical School, Boston, Massachusetts, United States of America; 4 College of Life Sciences, Southwest University, Chongqing, China; Virginia Commonwealth University, United States of America

## Abstract

Milk fat globule factor-E8 (MFG-E8) has been regarded as a key factor involved in the phagocytosis of apoptotic cells. We induced a lentivirus into the microglial cells for the augmentation or abrogation of MFG-E8 expression in mouse microglial cells, and investigated phagocytosis of phosphatidylserine tagged human red blood cells (hRBCs) in co-cultures. Increased MFG-E8 levels were associated with a significant increase in phagocytic activity compared to the controls. Conversely, phagocytosis dramitically decreased due to the abrogation of MFG-E8. In addition, the expression of the inflammatory cytokines, TNF-α and IL-1β, also increased or decreased in the microglial cells with the augmentation or abrogation of MFG-E8, respectively. Our findings indicate that the enhanced expression of MFG-E8 could increase phagocytosis of apoptotic cells; conversely, the rate of phagocytosis and the expression of inflammatory cytokines decreased when MFG-E8 expression was knocked down. Our results confirm that MFG-E8 plays an important role in phagocytosis, and possibly serves as an essential signal molecule for microglial cells.

## Introduction

Apoptotic cells and potential toxic materials are the result of degenerative cell processes [1]–[3]. The efficient removal of apoptotic material plays an important role in protecting the surrounding tissue from damage due to the release of proteins from the dying cells [4]. Microglial cells, which are macrophage-like cells in the central nervous system, have been regarded as a typical representative cell of the immune system that possess this neuroprotective function [5]. Previous studies confirmed that activated microglial cells not only produce cytotoxic factors that can cause injury to neurons, but also engulf apoptotic cells and thus keep the microenvironment of the surrounding tissue healthy [5]. These studies mainly focused on the accumulation of microglial cells and associated proinflammatory cytokines during the process of neurodegenerative diseases [5], [6]. But there has been insufficient investigation as to the mechanism(s) and process of microglial cell phagocytosis, or how this activity is regulated.

Increasing evidence obtained from macrophage experiments suggests that MFG-E8 can recognize phosphatidylserine (PS) as an “eat me” signal expressed on the outer membranes of apoptotic cells. After recognizing the PS signal, MFG-E8 then works as a bridge to attach the phagocytic cell to the apoptotic cell, which then triggers a subsequent signaling cascade response and stimulates the phagocytic process, allowing the phagocytes to destroy the dying cell. Some studies reported that the mechanism of PS-triggered phagocytosis includes the ability of phagocytes to locate and recognize the externalized PS on apoptotic cells either directly through a PS receptor [7], or through other bridging proteins [8], [9]. The macrophage-like ability of microglial cells may depend on MFG-E8 to recognize and attach to PS on apototic cells [10].

We propose that MFG-E8 plays a key role in inducing the phagocytic behaviour of microglial cells in response to apoptotic cells, but details of the interaction remain to be clarified. Fuller et al. quantified the production of MFG-E8 in the BV-2 microglial cell line and the function of this protein in the recognition and engulfment of apoptotic neurons induced by UV light exposure [11]. However, it is still unclear whether or not the phagocytosis was triggered by a unique PS externalization, because UV light exposure can directly induce cell apoptosis or death, which makes it difficult to accurately study PS externalization. We induced PS externalizaiton in hRBCs to evaluate the role of MFG-E8 in the process of phagocytosis. We hypothesized that the augmentation or abrogation of MFG-E8 activity in mouse microglial cells would dramatically affect phagocytosis of hRBCs with externalized PS.

Previous studies on the function of macrophages or microglial cells indicated that engulfment mediated by MFG-E8 not only sequesters potentially harmful cellular products from the surrounding tissue, but also suppresses proinflammatory cytokines thereby creating an anti-inflammatory environment that additionally protects the tissue [12]–[14]. In central nervous system, some studies indicated that microglial cells can phagocytize viable cells, which is called primary phagocytosis [15], [16]. Therefore, interruption any components in the PS/MFG-E8 mediated pathway can rescue healthy neurons [12]–[14]. However, it is unclear whether or not microglial phagocytosis mediated by MFG-E8 can affect inflammation. Thus, we also studied the inflammatory responses of the mouse microglial cells during the process of phagocytosis.

## Materials and Methods

### Materials and Ethics

This study was approved with a written consent by the IACUC of the Southwest Hospital, Third Minitary Medical University, Chongqing, China. Mice were supplied by the Animal Care Center of Southwest Hospital. All experiments were performed in accordance with the association for Research in Vision and Ophthalmology Statement for the Use of Animals in Ophthalmic and Vision Research. hRBCs were obtained from blood donors at the Southwest Hospital and all procedures were approved by the Human Subjects Committee of Southwest Hospital. The study protocol adhered to the Declaration of Helsinki for research involving human subjects. The participants provided their written consents and approved the consent procedure for our studies.

### Preparation of Phosphatidylserine and Oxidized Phosphatidylserine

Phosphatidylserine (PS, 1-Palmitoyl-2-arachidonyl-3-phosphatidylserine, Avanti-Polar Lipids, Alabaster, AL, USA) was dissolved in chloroform and then dried under nitrogen. The precipitate was dissolved in phoshate buffered saline (PBS) to make a PS stock solution at a concentration of 10 mM. An appropriate amount of 2,2-azobis [2-amidinopropane]-dihydrochloride (100 mM, Sigma, Shanghai, China) was mixed with the PS solution (volume ratio 1∶1) and incubated at 37°C for 4 hr. After the incubation, methyl alcohol and chloroform (volume ratio 2∶1) were used to extract oxidized PS. Oxidation was determined by measuring the absorbance of hydroperoxides with conjugate dienes (ultraviolet spectroscopy at 234 nm) [17]. The analysis indicated a 30∶70% mixture of oxidized and nonoxidized PS, and this mixture of PS species was called PSox [18]. The lipid mixture was vortexed and sonicated for 3 min on ice before being used to induce PS externalization.

### Isolation and Culture of Mouse Microglial Cells

Newborn (postnatal day 1–3 ) C57BL/6J mice were euthanized with a overdose of Ketamine and Xylazine (Wedochem, Jinan, China) at a dosage of 120 mg/kg and 20 mg/kg in saline, respectively. The anesthesic mixture was injected subcutaneously using a 27G needle. Mouse microglial cells were isolated from the brains using a modification of procedure given by Giulian and Baker [19]. In brief, the brain was immediately dissected free of skull and dissociated by trituration in 0.25% trypsin (Sigma, Shanghai, China) in 1× PBS. Cells were plated in 75 cm^2^ plastic flasks containing 10 ml DMEM/F12 medium (volume ratio 1∶1, Invitrogen, CA, USA) and cultured in an incubator (37°C, 5% CO_2_). The culture medium was changed every two days. On day 14, the mixed glial cultures were washed with DMEM/F12 medium, and then the flasks were shaken for 2 hr (240 rpm at 37°C) to purify microglial cells. The astroglia remaine adherent to the flasks thus this process can efficiently remove contaminating astrocytes. The suspension was replated in 75 cm^2^ plastic flasks with 10 ml DMEM/F12 and cultured for a further incubated again (37°C, 5% CO_2_) afterwhich for 3 hr. The oligodendroglia were removed by gently shaking the flasked at room temperature. Subsequently, the strongly adherenting microglial cells were subsequently dislodged released from the flasks by vigorous shaking in DMEM/F12 medium plus with 0.25% trypsin. The cell suspension was rewashed and seeded into culture flasks and incubated at 37°C with 5% of CO_2_ for 2 hr. The attached cells were removed by trypsinization and were seeded onto new plates and cultured in an incubator (37°C with 5% of CO_2_) for use in later experiments.

Surface expression of CD11b on microglial cells was checked by flow cytometry [20]. Briefly, cells were stained with fluorescein isothiocyanate (FITC)-conjugated mouse anti-CD11b (1∶200, BD Bioscience, CA, USA). Fluorescence-activated cell sorting (FACS) was used to detect the microglial phenotype.

Double staining for mouse anti-CD11b (1∶400, Chemicon, CA, USA) and rabbit anti-MFG-E8 (1∶200, Santa Cruz Biotechnology, CA, USA) and confocal microscopy was also performed. Cells were permeabilized by 0.1% Triton-X 100 during the normal staining process. Since extracellular MFG-E8 staining is detergent sensitive [13], [14], [21], it was therefore necessary to ensure that MFG-E8 was externalized to the cell surface. Thus, cells were also double labelled with CD11b and MFG-E8 using a non-permeabilized procedure [13], [14], [21]. FITC conjugated affiniPure goat anti-mouse IgG (1∶200) and Cy3-conjugated affiniPure goat anti-rabbit IgG (1∶200, both from Jackson ImmunoResearch, PA, USA) were used as secondary antibodies, respectively. MFG-E8 and CD11b were both localised to the sympatric on the cell surface. This finding coincided with the result of the staining on permeabized microglial cells, indicating that both methods show the externalization of which means that MFG-E8 externalizated on the surface of the microglial cells.

In order to identify the phagocytotic characteristics of cultured microglial cells, green fluorescence labeled microbeads (0.02 µm in diameter, Invitrogen, NY, USA) were introducted to the microglial cells culturing for 12 hr (37°C with 5% of CO_2_). Excessive microbeads were removed by 1× PBS washes containing 1 mM MgCl_2_ and 0.2 mM CaCl_2_.

### Isolation and Culture of hRBCs

We used hRBCs to examine the phagocytic behavior of microglial cells due to the ease of making microscopic observations and removes the necessity for fluorescent markers to label the cells. hRBCs were isolated from the blood samples with EDTA to prevent clotting, centrafuged at 1,500 rpm for 10 min, then washed three times with 1× PBS. Cells were resuspended in 1× PBS with 0.1% sucrose (volume ratio 1∶4) and the hRBC stored at 4°C as a stock solution.

### PS Externalization in hRBCs

An appropriate volume of hRBC stock solution was added to N-ethylmaleimide (NEM, Sigma, Shanghai, China) to make a solution with a NEM conconcentration of 20 µM or 30 µM. The mixture was incubated at 37°C for 10 min or 30 min and then centrifuged (1,900 rmp, 10 min) to collect the sediment. After three washes in PBS, the sediment was resuspended PBS and mixed with PS or PSox for 30 min at 37°C (PS or PSox concentration was adjusted to 20 µM in the mixture). The solution was contrifuged for 10 min (1,500 rmp), the supernatant were removed and the hRBCs were washed with 1× PBS (10 ml). PS-hRBCs were then used to assess the phagocytic activity of the microglial cells (see below). After treatment with NEM and integration with PS or PSox, the PS-hRBCs translocated the PS from the inner face to the outer surface of the plasma membrane of the cell (referred to as PS externalization in the present study). Cell surface PS can be easily detected by flow cytometry after labelling with a fluorescent conjugate of Annexin V-FITC, which is a specific protein with a high affinity for PS, thus confirming PS externalization on hRBC cells.

### Phagocytosis of hRBCs by Microglial Cells

Microglial cells were seeded into 6-well (2 × 10^5^ cells/well) and cultured overnight. Normal hRBCs or PS-hRBCs were resuspended in DMEM medium at a 0.1% concencentration. The hRBCs and PS-hRBCs were pre-labelled using a red fluorescence PKH kit (Sigma, Shanghai, China) according to the manufacturers instructions, and then added the wells at ratios between 1∶50–1∶100 (microglial cells vs hRBCs). The plates were incubated for 2 hr (37°C, 5% CO_2_), the medium removed and the plates washed three times with PBS (37°C). The phagocytosis of hRBCs or PS-hRBCs was examined using an inverted fluorescence microscope (Olympus BX41, Japan). Four fields (100×) in each well were randomly selected. Phagocytic rate was determined by counting the number of microglial cells containing the phagocytized hRBCs divided by the total number of microglial cells in each image.

Immuno-fluorescent staining was used to provide further confirmation of the phagocytic activity and internalization of hRBCs. Microglial cells and pre-labeled hRBCs using PKH kit were co-incubated in chamber slide (Lab-Tek Chamber Slides, Thermo) for 2 hr. Then cells were fixed and FITC conjugated mouse anti-CD11b (1∶200) was used to label the microglial cells. The cells were then examined using confocal microscopy.

### Transfection of Microglial Cells by Plasmids

Overexpression and knockdown of MFG-E8 in microglial cells was performed by transfection of a lentivirus [22]–[25]. For overexpression, a lentiviral vector system obtained from Genechem (Shanghai, China) was used as a template to amplify the MFG-E8 gene with site-directed mutagenesis. The resulting PCR product was digested with *Age*I, purified and cloned into the lentiviral vector pGC-FU (Genechem) to generate pGC-FU-MFG-E8. Meanwhile, pGC-FU vector was used as a negative control. For knockdown of MFG-E8, siRNA (5′-GCAGCTACAAGACATGGAA-3′) was designed by selecting the appropriate sequence from the MFG-E8 complete mRNA. The cDNA of double stranded shRNA oligo was cloned into pFU-GW lentivirus vector (Genechem) using the *Eco*R I and *Xho* I restriction enzyme, and generated pFU-GW-RNAi-MFG-E8. The recombinant plasmid was verified by DNA sequencing. A negative control shRNA unrelated to MFG-E8 sequence was used as a control. Human embryonic kidney (HEK) 293T cells were transfected with the packaging plasmids (pHelper 1.0 and pHelper 2.0) and lentiviral vector pFU-GW-MFG-E8 using lipofectamine 2000 (Invitrogen). The procedure of lentivirus production in HEK 293T cells was as previously described [22]–[25]. Infectious lentivirus vectors were harvested at 48 hr post-transfection, centrifuged to get rid of cell debris, and then filtered through 0.22 cellulose acetate filters. The infectious titer was determined by realtime qRT-PCR for pGC-FU-MFG-E8 and limiting dilution analysis for pFU-GW-RNAi-MFG-E8 in 293T cells [26]. The virus titers were at the range of 10^9^ transducing units/ml medium, and these viruses were kept at −80°.

Microglial cells (2 × 10^5^ cells/well) were seeded in 6-well plates with DMEM/F12 medium one day before the transfection. For lentiviral infection, lentivirus was added to transduction medium at a multiplicity of infection of 40 (Opti-MEM, GIBCO, Shanghai, China). Twenty-four hours after lentiviral infection, transduced cells were washed in 1× PBS and DMEM/F12 medium was added to wells. On day 3 post-infection, the cells were analyzed using confocal microscopy. On day 9, cells were collected, protein and total mRNA were extracted respectively (see below). The same protocol was used for the transfection of other plasmids.

### Evaluation of mRNA Expression by qRT-PCR

qRT-PCR was used to test the mRNA expression of MFG-E8 and inflammatory cytokines (TNF-α and IL-1β). Microglial cells were seeded in 24-well plates (5 × 10^4^ cells/well) for 24 hr. Normal RBCs and PS-hRBCs suspension were added to the wells and co-cultured (37°C, 5% CO_2_) for 2 hr. The cultures were washed three times with PBS (37°C) and then the cells in each plate collected and total mRNA extracted (RNeasy Mini kit, Qiagen, ML, USA) for cDNA synthesis (SuperScript III First-Strand Synthesis SuperMix, Invitrogen, CA, USA) and qRT-PCR. The procedures of mRNA extraction and cDNA synthesis followed those provided by the manufacturers and the primers are listed in Table 1. The procedure for qRT-PCR included 5 min at 99°C, followed by 40 cycles of 15 s at 94°C, 30 s at 59°C, and 45 s at 72°C (Roche LC480, Roche Applied Science). Expression (evaluated as fold change for each target gene) was normalized to ß-actin following the well-established delta-delta method [27], [28]. Data are presented as fold change over the control (i.e. microglial cells incubated with normal hRBCs). All assays were performed in triplicates. In addition, a non-template control was included in the experiment to estimate the DNA contamination of isolated RNA and reagents.

**Table 1:** Summary of the primers for qRT-PCR.

Genes	Primers	Sequences
MFG-E8	Forward	AGATGCGGGTATCAGGTGTGA
	Reverse	GGGGCTCAGAACATCCGTG
TNF-α	Forward	CCCCTCAGCAAACCACCA
	Reverse	GGCAGCCTTGTCCCTTGA
IL-1β	Forward	TGGGAAACAACAGTGGTCAGG
	Reverse	CCATCAGAGGCAAGGAGGAA
β-actin	Forward	GCGTGACATCAAAGAGAAGC
	Reverse	AGCACTGTGTTGGCATAGAG

### MFG-E8 Expression in Transfected Microglial Cells and Western Blot Analysis

Western blot assays were used to assess MFG-E8 expression in transfected microglia. Briefly, the transfected cells were completely lysed in 10 mM Tris-HCl (pH 7.6) containing 2 mM Na orthovanadate (pH 7.6), 0.2 mM PMSF, 2 mg/ml leupeptin, 2 mg/ml aprotinin and 1 tablet of Complete Mini (Roche Diagnostic, IN, USA). An equal amount of protein per lane was loaded into a 4–20% Bis-Tris gel (Invitrogen), which was transferred to a 0.2 mm nitrocellulose membrane (Invitrogen). The membrane was incubated overnight at 4°C with rabbit anti-MFG-E8 (1∶300), washed three times in 1× PBST (PBS with 0.05% Tween 20 (Sigma, Shanghai, China)) for 40 min, and then incubated with secondary antibodies (goat anti-rabbit HRP and HRP-conjugated GAPDH; R&D System, Minneapolis, MN, USA) at a dilution of 1∶10,000 in TBST for 1 hr at room temperature. The membrane was washed three times in TBST (40 min) and then the chemiluminescent signals on the membrane captured on film (Thermo Scientific, Rockkford, IL, USA). All experiments were repeated three times.

### Statistical Analysis

All statistical tests were performed using SPSS 11.0. Independent Samples t-test were used to compare the differences in the MFG-E8 expression between groups after transfection. All data are presented as the mean ± SD and comparisons with P≤0.05 were considered significant.

## Results

### Phagocytic Model and Evaluation PS Externalization in hRBCs

**Figure 1:**
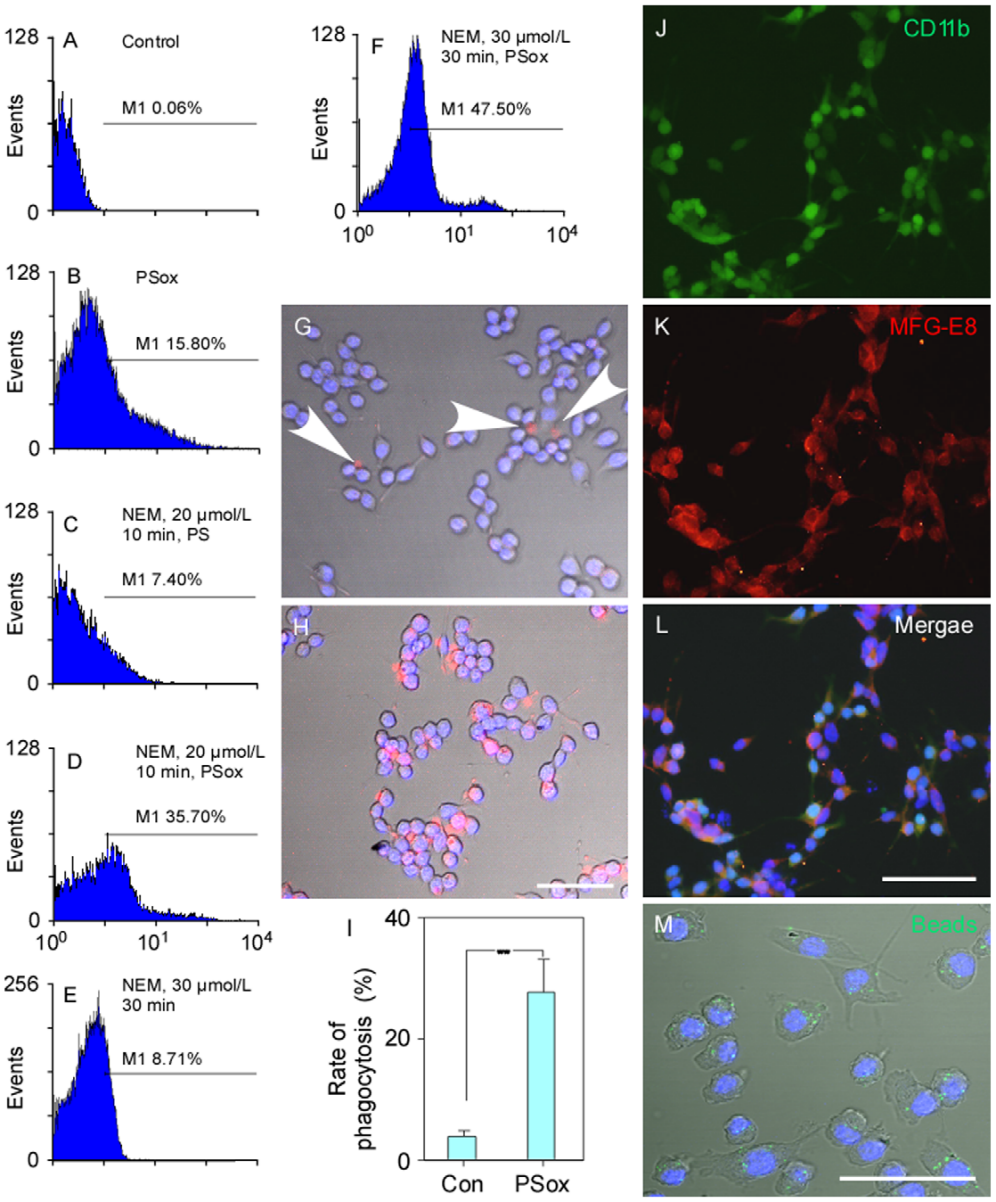
The detection of phosphatidylserine externalization in hRBCs labelled with Annexin V FITC fluorescence (flow cytometry) (A–F) and the phagocytosis of these cells by mouse microglial cells (G–H). (A–F) PS externalization was (35.70%) most successful with incubation for 10 min with 20 µM NEM and then incubated with 20 µM PSox for 30 min. Although, 47.50% of hRBCs showed PS externalization after being incubated for 30 min with 30 µM NEM and then 30 min with 20 µM PSox for 30 min, cells were fragile and considered unsuitable for use. (G) Prelabled normal hRBCs were very rarely recognised by microglial cells, but (H) phagocytosis was common for prelabeled PSox-hRBS cells. (I) The hRBSCs phagocytosis rate was significantly higher for PSox-treated cells. Immunoflurescent staining indicated that the cultured cells expressed CD11b and MFG-E8 (J–L). (M) The majority of cultured microglial cells can incorporate microbeads (green) suggesting that they have the ability of non-specific phagocytosis. Scale bars: 50 µm for G–H, 100 µm for J–M. ** *P*<0.01. Abbreviations: Con, control; PSox: mixture of oxidized and nonoxidized phosphatidylserine; hRBCs, human red blood cells.

Under normal physiological situations, PS was usually found on the inner side of the cell membrance. PS externalization in normal hRBCs after labelling with Annexin V-FITC was very rarely detected by flow cytometry (0.06%) (Figure 1A). The externalization rate increased to 15.80% when hRBCs were incubated with PSox (Figure 1B) or 7.40% (Figure 1C) when pre-treated (for 10 min in 20 µM NEM) hRBCs were cultured with PS. In contrast, the externalization rate increased to 35.70% when pre-treated hRBCs were incubated with PSox (Figure 1D). Increasing the pre-treatment time and concentration in NEM (30 min, 30 µM) without PS incubation did not significantly increase externalization (8.71%; Figure 1E vs. 1C). When hRBCs was pre-treateated in 30 µM NEM for 30 min followed by PSox, externalizatgion was higher (47.50%) than that at lower NEM concentrations (Figure 1F vs. 1D). However, hRBCs appeared to become fragile at these concentrations and incubation times. Therefore, in the following experiments a regime 20 µM NEM,10 min with PSox was used to induce stable PS externalization.

After inducing stable PS exernalization in hRBCs they were co-cultured with microglial cells. Only 3.80±1.00% of the normal hRBCs were recognized and phagocytosed by the microglial cells (Figure 1G), compared to 27.50±5.40% after the hRBCs were treated with NEM and PSox (Figure 1H), which was a significant increase in the phagocytic rate (n = 4, *P*<0.01; Figure 1I).

FACS showed a population of 91–93% CD11b positive cells, which were regarded as microglial cells. In addition, immunoflurescent staining verified that the cultured cells expressed CD11b and MFG-E8 (Figure 1J–L). MFG-E8 and CD11b were both localised to the cell surface and labeling was similar to that seen on permeabized microglial cells (data not shown), confirming the presence of microglial cells containing a possible bridge to attach to the hRBCs, i.e. MFG-E8. Furthermore, 98% of microglial cells contained microbeads (Figure 1M), indicating that they have the ability for non-specific phagocytosis. All of these findings confirmed that the microglial cells were suitable as a phagocytic model for the modified expression of MFG-E8 in the following experiments.

### Evaluation of MFG-E8 Overexpression and Knockdown

**Figure 2:**
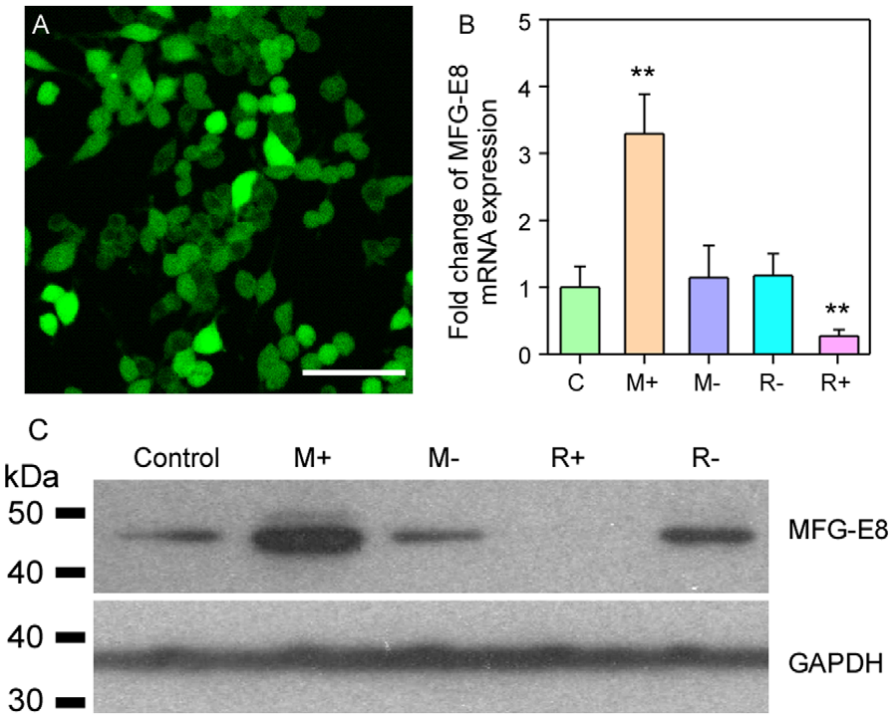
Overexpression and knockdown of MFG-E8 in microglial cells after transfection with a lentivirus. Four different transfections were used: M+, MFG-E8 overexpression using pGC-FU-MFG-E8; M-, MFG-E8 negative control for overexpression using the pGC-FU vector; R+, Knockdown expression using pFU-GW-RNAi-MFG-E8; R- negative control for knockdown expression using the pFU-GW Vector. (A) The transfection rate was over 80% in all experiments. (B) qRT-PCR analysis indicated that pGC-FU-MFG-E8 significantly enhanced microglial MFG-E8 expression, and pFU-GW-RNAi-MFG-E8 effectively knocked it down. The negative controls had no effect. (C) Western blot analysis of the cell lysate from the transfected microglial cells confirmed that MFG-E8 expression (about 47 kDa) was significantly enhanced or knocked down. Scale bar: 50 µm. ** *P*<0.01.

Fluorecent micorscopy indicated that the viral vectors were successfully transferred to most cells (over 80%; Figure 2A). qRT-PCR analysis indicated that pGC-FU-MFG-E8 significantly enhanced MFG-E8 expression, and conversely, pFU-GW-RNAi-MFG-E8 effectively knocked down MFG-E8 expression compared with nontransfected microglial cells and negative controls (*P*<0.05; M+ and R+ vs. all others, Figure 2B). The negative controls had no effect on MFG-E8 expression compared with nontransfected controls (both *P*>0.05; M- and R- vs. C, Figure 2B).

The cell lysate from the transfected microglial cells was used to analyze the expression of MFG-E8. Western blot analysis indicated that MFG-E8 expression (about 47 kDa) significantly increased in pGC-FU-MFG-E8 transfected microglial cells (M+, Figure 2C). In contrast, the expression was dramatically reduced after pFU-GW- RNAi-MFG-E8 transfection (R+, Figure 2C). MFG-E8 expression was found to be similar for the negative transfection and controls (M-, R- and C, Figure 2C).

### Phagocytic Properties of Microglial Cells

**Figure 3:**
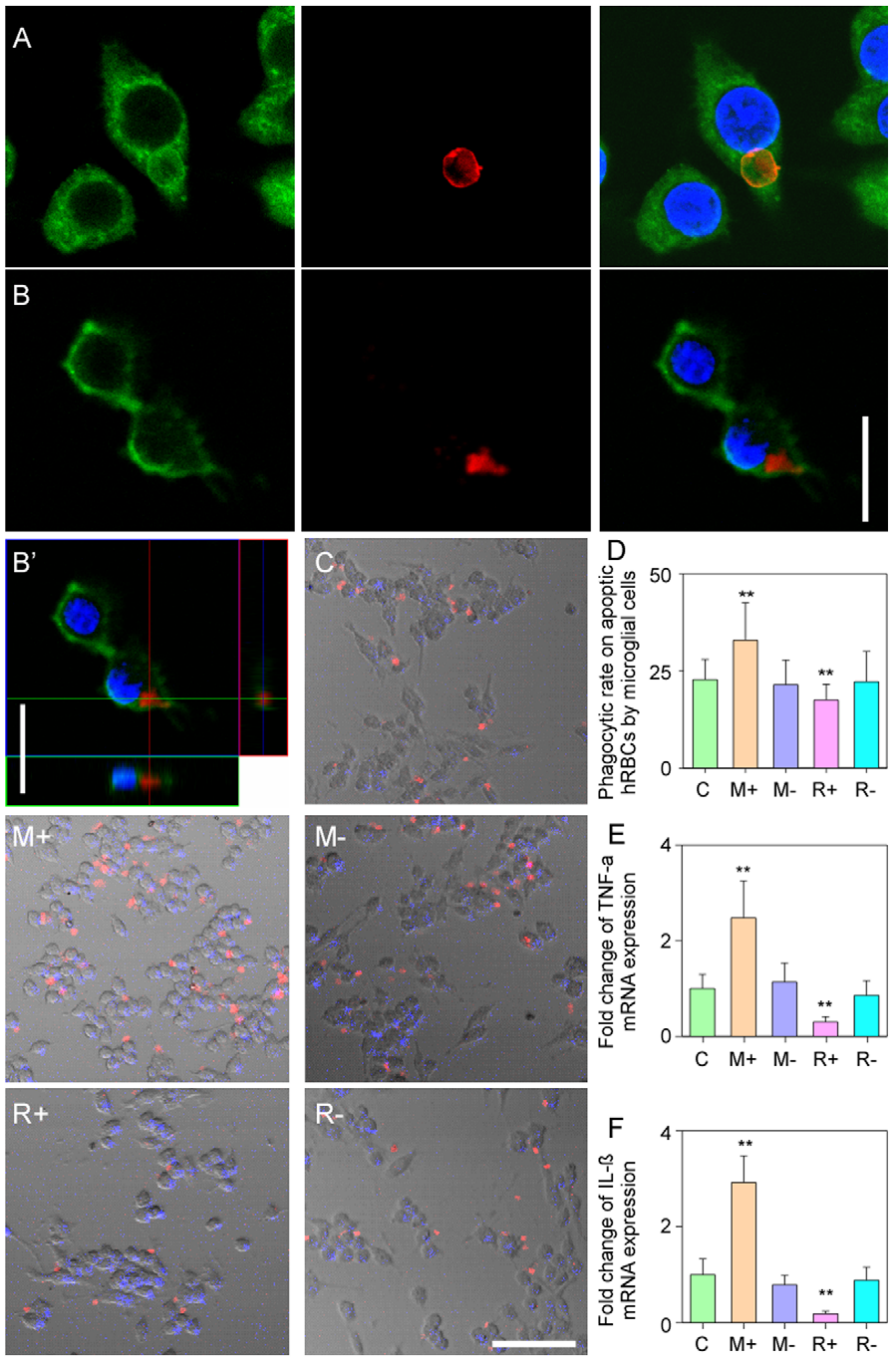
The phagocytosis of PS-hRBCs and mRNA expression of TNF-α and IL-1β by microglial cell co-cultures. (A) Representative confocal images showing the phagocytosis of of prelabeled PSox-hRBCs (red). (B–B′) The internalization of the PSox-hRBCs (red) material into the microglial cell (labeled with GFP) can be seen clearly in the confocal z-stacked images. (C) Control microglial cells without transfection; M+, MFG-E8 overexpression of pGC-FU-MFG-E8; M-, pGC-FU transfection; R+, blocked MFG-E8 expression- pFU-GW-MFG-E8; R-, nonsense sequence in the pFU-GW. (D) Overexpression of MFG-E8 in microglial cells increased the phagocytosis of PS-hRBCs; phagocytosis rate dramatically decreased in knocked downs. (E–F) The mRNA expression of TNF-α and IL-1β increased in the MFG-E8 up-regulated microglial cell, but significantly decreased when MFG-E8 was knocked down. Scale bars in A–B: 20 µm; C–R-: 50 µm; ** *P*<0.01.

Confocal microscopy and Z-stacking confirmed that the microglial cells could effectively “grasp” and phagocytize and internalize hRBCs (Figure 3A and 3B–B′). Four different transfection experiments were used to evaluate the phagocytosis of hRBCs. The phagocytic rate was about 22.8% for the nontransfected microglial cells (Figure 3C). The phagocytic rate of microglial cells with a high MFG-E8 expression was significantly higher (32.9%, Figure 3M+) than negative controls (21.5%; Figure 3M−, *P*<0.01, Figure 3D). In the MFG-E8 knock-down cells, phagoctic rate significantly decreased to 17.5% (Figure 3R+) compared with that of other transfected microglial cells or the nontransfected controls (Figure 3R- and Figure 3D, all *P*<0.01). In addition, a significant difference was not found between the controls (Figure 3C, 3M- and 3R-; *P*>0.05, Figure 3D). Therefore, MFG-E8 plays a significant role in recognizing apoptotic cells.

Interestingly, differneces in the mRNA expression of the typical inflammatory cytokines, TNF-α and IL-1β, appear to coincide with the phagoctic rate following the different transfection protocols. Specifially, microglial cells with an overexpression of MFG-E8 had significantly high expression of TNF-α and IL-1β (Figure 3E–F, *P*<0.01); conversely, the lowest expression of TNF-α and IL-1β mRNA was found in microglial cells with significantly reduced MFG-E8 expression (Figure 3E–F, *P*<0.01). There was no statistical difference among the controls with respect to cytokine expression (Figure 3E–F, all *P*>0.05).

## Discussion

One of the initial changes in the process of apoptosis is the translocation of PS from the inner membrane to the outer surface of a cell. PS is most widely studied “eat-me” signal, although others include changes in the surface charge of glycoproteins and lipids (owing to the addition of sugars), the binding of thrombospondin or the complement component C1q to the apoptotic cell surface, the expression of intercellular adhesion molecule 3, and an oxidized low-density lipoprotein-like moiety [29]. In order to induce the phagocytic behavior of microglial cells, we tagged hRBCs with an externalized PS “eat-me” signal, which subsequently trigered the phagocytic process. Although previous studies have used different ways to induce phagocytosis, such as UV light exposure [11] or dexamethasone treatment [8], these methods may cause cell death or trigger some other cellular membrane morphological change. Therefore, we used NEM and PSox to encapsulate hRBCs to induce the apoptotic signals. In the execution of the process, PS underwent a statistically significant and pronounced oxidation [17] and NEM specifically enhanced PS exposure [30], while maintaining the cellular integrity in more than 95% of the hRBCs. This externalisation of PSox significantly increased the phagocytosis of the hRBCs comparing to previous studies.

MFG-E8, containing a PS binding domain, has been comfirmed as a binding bridge between apoptotic cells and integrins on the surface of macrophages. Some studies indicated that MFG-E8 secreted from activated macrophages, specifically binds to externalized PS on apoptotic cells and promotes their subsequent engulfment [8], [31]. In the central nervous system, further study showed that MFG-E8 increased the phagocytosis of apoptotic neurons by microglial cell lines [11]. According to our findings, the phagocytic behavior of microglial cells can be significantly modified by enhancing or knocking down the expression of MFG-E8, i.e. phagocytic rate significantly increased in accordance with an increase in MFG-E8 expression in the microglial cells, and conversely, decreased when MFG-E8 expression was knocked down. Recent studies showed that inflamed microglial cells release reactive oxygen or nitrogen species, which induced reversible PS exposure on the surface of viable neurons [14], [16], [32] These neurons were phagocytosed and thus killed when they interacted with microglial cells [15], [16]. And this process was mediated by a pathway consisting of neuronal PS exposure, recognition of PS by the bridging molecule MFG-E8, and MFG-E8 binding to the vitronectin receptor [14], [16], [32]. In our study, although we did not test whether up-regulation of MFG-E8 in microglial cells can induce PS exposure to hRBCs, the increase of MFG-E8 expression on the surface enhanced the phagocytosis by microglial cells.

An *in vivo* study suggested that MFG-E8 could lead to an anti-inflammatory response, because the study showed that TNF-α and IL-1β were clearly downregulated in mouse macrophages [33], thus the presence of MFG-E8 secreted by macrophages effectively reduces the inflammatory response and leads to a decrease in extracellular inflammatory factors. Similarly, Miksa et al. found that an increase in MFG-E8 through exosome treatment [34], increased the clearance of apoptotic cells and reduced levels of inflammatory factors such as TNF-α in the rat. Exposure to apoptotic signals can quickly activate resting microglial cells to play their roles in tissue maintenance, such as inflammatory activation. There is no direct evidence to show the production of the inflammatory factors during phagocytosis or while microglial cells work as anti-inflammation agents *in vivo*.

ur *in vitro* study shows that TNF-α and IL-1β expression was significantly higher in microglial cells with a higher expression of MFG-E8. These findings are not consistent with the previous studies. A previous study indicated that microglial TNF-α expression increased in outer nuclear layer of the *rd* mouse retina at a time when microglial cells were activiated [35]. Conversey, as phagocytosis progresses and MFG-E8 levels depleted, the level of inflammatory factors in extracellular microenvironment will also be continuously reduced. We suggest that these differences are due to differences in the inflammatory responses of macrophages vs. microglial cells. The expression of MFG-E8 is altered in subjects with autoimmune diseases, such as systemic lupus erythematosus (SLE) [36]. Specifically, a deficiency of MFG-E8 production has been reported to develop SLE in mice, leading to an impaired clearance of apoptotic cells [8], [31]; however, MFG-E8 serum levels in SLE patients were higher than in healthy individuals [37], [38]. Therefore, as Aziz et al. pointed out that the expression of inflammatory factor from different subjects could lead to the noncoincidence between different studies [36]. Interestingly, a recent study indicates that inhibition of microglial phagocytosis significantly prevent inflammatory neuronal death *in vitro* [14], which is defined as primary phagocytosis [14], [15]. This study found that inhibition of any step in primary phagocytosis pathway, such as reducing PS exporsure or blocking MFG-E8 expression, rescued over 90% of neurons [14], [16]. Those findings indirectly indicated that modification of MFG-E8 expression can lead to the variation of phagocytosis by microglial cells, which is in accordance with our findings.

### Conclusions

We are the first to successfully devise a novel method to augment or abrogate MFG-E8 expression in microglial cells to investigate the phagocytosis of hRBCs. Our findings indicate that the enhanced expression of MFG-E8 is able to significantly increase phagocytosis, which is very important for maintaining healthy tissue; in contrast, the phagocytic rate decreased when the expression of MFG-E8 was abrogated. Therefore, our results confirm that MFG-E8 plays an important role in the phagocytic function of microglial cells. These findings could provide a basis for future studies on the clinical treatment of some diseases casued by phagocytic disorders of the microglial cells.

## References

[1] Mochizuki H, Goto K, Mori H, Mizuno Y (1996) Histochemical detection of apoptosis in Parkinson’s disease. Journal of the Neurological Sciences 137: 120–123. 8782165 10.1016/0022-510x(95)00336-z

[2] Thomas LB, Gates DJ, Richfield EK, O’Brien TF, Schweitzer JB, et al (1995) DNA end labeling (TUNEL) in Huntington’s disease and other neuropathological conditions. Experimental Neurology 133: 265–272. 7649231 10.1006/exnr.1995.1029

[3] Zhang L, Kokkonen G, Roth GS (1995) Identification of neuronal programmed cell death in situ in the striatum of normal adult rat brain and its relationship to neuronal death during aging. Brain Research 677: 177–179. 7606465 10.1016/0006-8993(95)00197-x

[4] Savill J, Fadok V (2000) Corpse clearance defines the meaning of cell death. Nature 407: 784–788. 11048729 10.1038/35037722

[5] Saijo K, Glass CK (2011) Microglial cell origin and phenotypes in health and disease. Nature Review Immunology 11: 775–787. 10.1038/nri3086 22025055

[6] Rock RB, Gekker G, Hu S, Sheng WS, Cheeran M, et al (2004) Role of microglia in central nervous system infections. Clinical Microbiology Reviews 17: 942–964. 15489356 10.1128/CMR.17.4.942-964.2004 PMC523558

[7] Fadok VA, Bratton DL, Rose DM, Pearson A, Ezekewitz RAB, et al (2000) A receptor for phosphatidylserine-specific clearance of apoptotic cells. Nature 405: 85–90. 10811223 10.1038/35011084

[8] Hanayama R, Tanaka M, Miwa K, Shinohara A, Iwamatsu A, et al (2002) Identification of a factor that links apoptotic cells to phagocytes. Nature 417: 182–187. 12000961 10.1038/417182a

[9] Hall MO, Obin MS, Heeb MJ, Burgess BL, Abrams TA (2005) Both protein S and Gas6 stimulate outer segment phagocytosis by cultured rat retinal pigment epithelial cells. Experimental Eye Research 81: 581–591. 15949798 10.1016/j.exer.2005.03.017

[10] Leonardi-Essmann F, Emig M, Kitamura Y, Spanagel R, Gebicke-Haerter PJ (2005) Fractalkine-upregulated milk-fat globule EGF factor-8 protein in cultured rat microglia. Journal of Neuroimmunology 160: 92–101. 15710462 10.1016/j.jneuroim.2004.11.012

[11] Fuller A, Van Eldik L (2008) MFG-E8 regulates microglial phagocytosis of apoptotic neurons. Journal of Neuroimmune Pharmacology 3: 246–256. 18670887 10.1007/s11481-008-9118-2 PMC2832904

[12] Fadok VA, Bratton DL, Konowal A, Freed PW, Westcott JY, et al (1998) Macrophages that have ingested apoptotic cells in vitro inhibit proinflammatory cytokine production through autocrine/paracrine mechanisms involving TGF-β, PGE2, and PAF. The Journal of Clinical Investigation 101: 890–898. 9466984 10.1172/JCI1112 PMC508637

[13] Fricker M, Neher JJ, Zhao JW, Théry C, Tolkovsky AM, et al (2012) MFG-E8 mediates primary phagocytosis of viable neurons during neuroinflammation. The Journal of Neuroscience 32: 2657–2666. 22357850 10.1523/JNEUROSCI.4837-11.2012 PMC3312099

[14] Neher JJ, Neniskyte U, Zhao JW, Bal-Price A, Tolkovsky AM, et al (2011) Inhibition of microglial phagocytosis is sufficient to prevent inflammatory neuronal death. The Journal of Immunology 186: 4973–4983. 21402900 10.4049/jimmunol.1003600

[15] Fricker M, Oliva-Martín MJ, Brown GC (2012) Primary phagocytosis of viable neurons by microglia activated with LPS or Aβ is dependent on calreticulin/LRP phagocytic signalling. Journal of Neuroinflammation 9: 196. 22889139 10.1186/1742-2094-9-196 PMC3481398

[16] Neher JJ, Neniskyte U, Brown GC (2012) Primary phagocytosis of neurons by inflamed microglia: potential roles in neurodegeneration. Frontiers in Pharmacology 3: 27. 22403545 10.3389/fphar.2012.00027 PMC3288722

[17] Kagan VE, Gleiss B, Tyurina YY, Tyurin VA, Elenström-Magnusson C, et al (2002) A role for oxidative stress in apoptosis: oxidation and externalization of phosphatidylserine is required for macrophage clearance of cells undergoing Fas-mediated apoptosis. The Journal of Immunology 169: 487–499. 12077280 10.4049/jimmunol.169.1.487

[18] Tyurina YY, Serinkan FB, Tyurin VA, Kini V, Yalowich JC, et al (2004) Lipid antioxidant, etoposide, inhibits phosphatidylserine externalization and macrophage clearance of apoptotic cells by preventing phosphatidylserine oxidation. The Journal of Biological Chemistry 279: 6056–6064. 14630936 10.1074/jbc.M309929200

[19] Giulian D, Baker T (1986) Characterization of ameboid microglia isolated from developing mammalian brain. The Journal of Neuroscience 6: 2163–2178. 3018187 10.1523/JNEUROSCI.06-08-02163.1986 PMC6568755

[20] Roy A, Fung YK, Liu X, Pahan K (2006) Up-regulation of microglial CD11b expression by nitric oxide. Journal of Biological Chemistry 281: 14971–14980. 16551637 10.1074/jbc.M600236200 PMC1963414

[21] Oshima K, Aoki N, Kato T, Kitajima K, Matsuda T (2002) Secretion of a peripheral membrane protein, MFG-E8, as a complex with membrane vesicles. European Journal of Biochemistry 269: 1209–1218. 11856354 10.1046/j.1432-1033.2002.02758.x

[22] Dull T, Zufferey R, Kelly M, Mandel RJ, Nguyen M, et al (1998) A third-generation lentivirus vector with a conditional packaging system. Journal of Virology 72: 8463–8471. 9765382 10.1128/jvi.72.11.8463-8471.1998 PMC110254

[23] Kita-Matsuo H, Barcova M, Prigozhina N, Salomonis N, Wei K, et al (2009) Lentiviral vectors and protocols for creation of stable hESC lines for fluorescent tracking and drug resistance selection of cardiomyocytes. PLoS ONE 4: e5046. 19352491 10.1371/journal.pone.0005046 PMC2662416

[24] Škalamera D, Ranall MV, Wilson BM, Leo P, Purdon AS, et al (2011) A high-throughput platform for lentiviral overexpression screening of the human ORFeome. PLoS ONE 6: e20057. 21629697 10.1371/journal.pone.0020057 PMC3101218

[25] Zufferey R, Dull T, Mandel RJ, Bukovsky A, Quiroz D, et al (1998) Self-inactivating lentivirus vector for safe and efficient in vivo gene delivery. Journal of Virology 72: 9873–9880. 9811723 10.1128/jvi.72.12.9873-9880.1998 PMC110499

[26] Geraerts M, Willems S, Baekelandt V, Debyser Z, Gijsbers R (2006) Comparison of lentiviral vector titration methods. BMC Biotechnology 6: 34. 16836756 10.1186/1472-6750-6-34 PMC1534021

[27] Livak KJ, Schmittgen TD (2001) Analysis of relative gene expression data using real-time quantitative PCR and the 2^−ΔΔ*C*T^ method. Methods 25: 402–408. 11846609 10.1006/meth.2001.1262

[28] Schmittgen TD, Livak KJ (2008) Analyzing real-time PCR data by the comparative *C* _T_ method. Nature Protocols 3: 1101–1108. 18546601 10.1038/nprot.2008.73

[29] Ravichandran K (2011) Beginnings of a good apoptotic meal: the find-me and eat-me signaling pathways. Immunity 35: 445–455. 22035837 10.1016/j.immuni.2011.09.004 PMC3241945

[30] Marguet D, Luciani M-F, Moynault A, Williamson P, Chimini G (1999) Engulfment of apoptotic cells involves the redistribution of membrane phosphatidylserine on phagocyte and prey. Nature Cell Biology 1: 454–456. 10559991 10.1038/15690

[31] Hanayama R, Tanaka M, Miyasaka K, Aozasa K, Koike M, et al (2004) Autoimmune disease and impaired uptake of apoptotic cells in MFG-E8-deficient mice. Science 304: 1147–1150. 15155946 10.1126/science.1094359

[32] Neniskyte U, Neher JJ, Brown GC (2011) Neuronal death induced by nanomolar amyloid beta is mediated by primary phagocytosis of neurons by microglia. The Journal of Biological Chemistry 286: 39904–39913. 21903584 10.1074/jbc.M111.267583 PMC3220594

[33] Aziz M, Jacob A, Matsuda A, Wu R, Zhou M, et al (2011) Pre-treatment of recombinant mouse MFG-E8 downregulates LPS-induced TNF-α production in macrophages via STAT3-mediated SOCS3 activation. PLoS ONE 6: e27685. 22114683 10.1371/journal.pone.0027685 PMC3217009

[34] Miksa M, Wu R, Dong W, Das P, Yang D, et al (2006) Dendritic cell-derived exosomes containing milk fat globule epidermal growth factor-factor Viii attenuate proinflammatory responses in sepsis. Shock 25: 586–593. 16721266 10.1097/01.shk.0000209533.22941.d0

[35] Zeng HY, Zhu X, Zhang C, Yang LP, Wu LM, et al (2005) Identification of sequential events and factors associated with microglial activation, migration, and cytotoxicity in retinal degeneration in *rd* mice. Investigative Ophthalmology & Visual Science 46: 2992–2999. 16043876 10.1167/iovs.05-0118

[36] Aziz M, Jacob A, Matsuda A, Wang P (2011) Review: milk fat globule-EGF factor 8 expression, function and plausible signal transduction in resolving inflammation. Apoptosis 16: 1077–1086. 21901532 10.1007/s10495-011-0630-0

[37] Yamaguchi H, Fujimoto T, Nakamura S, Ohmura K, Mimori T, et al (2010) Aberrant splicing of the milk fat globule-EGF factor 8 (MFG-E8) gene in human systemic lupus erythematosus. European Journal of Immunology 40: 1778–1785. 20213738 10.1002/eji.200940096

[38] Yamaguchi H, Takagi J, Miyamae T, Yokota S, Fujimoto T, et al (2008) Milk fat globule EGF factor 8 in the serum of human patients of systemic lupus erythematosus. Journal of Leukocyte Biology 83: 1300–1307. 18303131 10.1189/jlb.1107730

